# Research Status of Agricultural Nanotechnology and Its Application in Horticultural Crops

**DOI:** 10.3390/nano15100765

**Published:** 2025-05-20

**Authors:** Xiaobin Wen, Zhihao Lin, Bin Sheng, Xueling Ye, Yiming Zhao, Guangyang Liu, Ge Chen, Lin Qin, Xinyan Liu, Donghui Xu

**Affiliations:** 1 College of Horticulture, Shenyang Agricultural University, Shenyang 110866, China; 15931120675@163.com (X.W.); s1756313189@163.com (B.S.); 2 State Key Laboratory of Vegetable Biobreeding, Institute of Vegetables and Flowers, Chinese Academy of Agricultural Sciences, Key Laboratory of Vegetables Quality and Safety Control, Ministry of Agriculture and Rural Affairs of China, Beijing 100081, China; zhihaol401@163.com (Z.L.); kazuakizhao@163.com (Y.Z.); chenge@caas.cn (G.C.); qinlin01@caas.cn (L.Q.); liuxinyan@caas.cn (X.L.); xudonghui@caas.cn (D.X.); 3 National Center of Technology Innovation for Comprehensive Utilization of Saline-Alkali Land, 8 Zhihui Road, Agricultural High Tech Industry Demonstration Zone, Yellow River Delta, Dongying 257347, China

**Keywords:** agricultural nanotechnology, horticultural products, gene delivery, growth and development regulation, identification and detection of pollutants, fresh-keeping of fruits and vegetables

## Abstract

Global food security is facing numerous severe challenges. Population growth, climate change, and irrational agricultural inputs have led to a reduction in available arable land, a decline in soil fertility, and difficulties in increasing crop yields. As a result, the supply of food and agricultural products is under serious threat. Against this backdrop, the development of new technologies to increase the production of food and agricultural products and ensure their supply is extremely urgent. Agricultural nanotechnology, as an emerging technology, mainly utilizes the characteristics of nanomaterials such as small size, large specific surface area, and surface effects. It plays a role in gene delivery, regulating crop growth, adsorbing environmental pollutants, detecting the quality of agricultural products, and preserving fruits and vegetables, providing important technical support for ensuring the global supply of food and agricultural products. Currently, the research focus of agricultural nanotechnology is concentrated on the design and preparation of nanomaterials, the regulation of their properties, and the optimization of their application effects in the agricultural field. In terms of the research status, certain progress has been made in the research of nano-fertilizers, nano-pesticides, nano-sensors, nano-preservation materials, and nano-gene delivery vectors. However, it also faces problems such as complex processes and incomplete safety evaluations. This review focuses on the horticultural industry, comprehensively expounding the research status and application progress of agricultural nanotechnology in aspects such as the growth regulation of horticultural crops and the quality detection and preservation of horticultural products. It also deeply analyzes the opportunities and challenges faced by the application of nanomaterials in the horticultural field. The aim is to provide a reference for the further development of agricultural nanotechnology in the horticultural industry, promote its broader and more efficient application, contribute to solving the global food security problem, and achieve sustainable agricultural development.

## 1. Introduction

In the context of the continuous growth of the global population and the exacerbating impact of climate change, ensuring the global supply of food security has emerged as a crucial issue that concerns the survival and development of humanity. It is predicted that by 2050, global food demand will increase by 70%, posing extremely severe challenges to agricultural production [1,2]. However, currently, the production of food and horticultural products faces various stresses, severely threatening the stability and sustainability of the food security supply.

**Figure 1:**
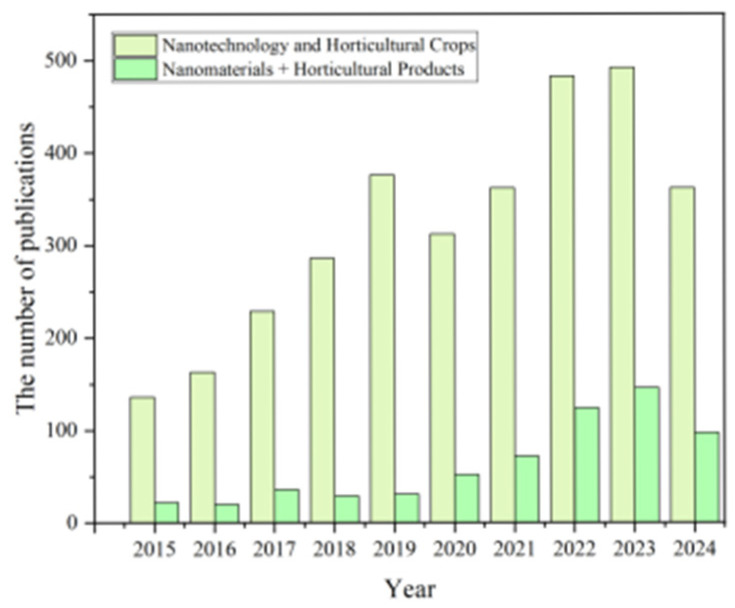
Publication trends of research papers in the field of nanomaterials, nanotechnology, horticultural crops and products in the past decade (data from Web of Science, with keywords “nanotechnology and horticultural crops” and “nanomaterials and horticultural products”) (statistical data as of December 2024).

After reviewing the literature on nanotechnology and horticultural crops, as well as nanomaterials and horticultural products over the past decade, it was found that the number of publications on horticultural products is relatively scarce (Figure 1). This article focuses on the applications of nanomaterials in horticulture. The aim is to provide references for the further development of agricultural nanotechnology in the horticultural industry, thereby promoting its broader and more efficient application.

During crop growth, biotic and abiotic stresses are important factors restricting the improvement of crop yield and quality [3]. In biotic stress, the rampant spread of diseases and pests, such as insects nibbling on crops and various diseases caused by pathogenic microorganisms, often leads to significant crop yield reduction or even complete crop failure [4,5]. In terms of abiotic stress, harsh environmental conditions such as drought, flood, high temperature, low temperature, and soil salinization interfere with the normal physiological and metabolic processes of crops, reducing the stress resistance of crops and thus affecting the yield [6,7].

At the same time, the extensive use of chemical substances (such as fertilizers and pesticides), although to some extent helpful for the prevention and control of diseases and pests and crop growth, has also triggered a series of negative effects. Currently, the annual consumption of chemical fertilizers and pesticides globally reaches as high as 400 million tons and 3 million tons, respectively, but the transportation and utilization efficiency of these agricultural chemicals is extremely low [8]. More than 50% of nitrogen (N) and 85% of phosphorus (P) are not absorbed by crops but are released into the environment, which seriously affects the biogeochemical cycle of elements [9]. The excessive application of fertilizers not only wastes resources but also leads to an imbalance of soil nutrients, destruction of soil structure, damage to the microbial community, and a continuous decline in soil fertility during the application process [10]. The abuse of pesticides, on the other hand, has caused the pesticide residues in agricultural products to exceed the standard. Moreover, due to the high solubility of pesticides, water sources will also be polluted. This not only endangers human health but also disrupts the ecological balance and affects biodiversity [11].

The post-harvest storage stage also has prominent problems. High-quality fruits and vegetables largely depend on good post-harvest treatment. Although there are already many methods to protect the quality and safety of fresh horticultural products, it is also important to conduct continuous research on the development of safer and more sustainable technologies [12]. Grains and horticultural products are extremely vulnerable to microbial contamination during the storage process, leading to phenomena such as mildew and rot, resulting in a large amount of wasted resources. In addition, the residues of pollutants in products, including pesticides, heavy metals, and organic pollutants, enter the human body through the food chain, and long-term accumulation poses a serious threat to health [11,13].

To address these challenges, the agricultural field urgently needs to make breakthroughs in several key areas. Firstly, in the breeding of resistant varieties, cultivating crop varieties with stronger resistance to diseases, pests, and abiotic stresses can ensure the healthy growth of crops from the source and reduce the dependence on chemical inputs, which is closely related to the development of gene editing technology using nanocarriers [14]. Genetic engineering methods have emerged in response to this need. These genetic engineering methods require the effective delivery of various biomolecules such as DNA, RNA, and proteins into plant tissues. To date, the most mature and commonly used methods to achieve this goal are Agrobacterium-mediated transformation, biolistic (gene gun) delivery, and protoplast transformation [14].

Developing green inputs, such as environmentally friendly fertilizers and pesticides, is an important way to reduce chemical pollution and achieve sustainable agricultural development [15]. Optimizing crop growth and development using nanoregulation technology can improve crop yield and quality [3]. Achieving accurate identification, detection, effective control, and removal of pollutants is the key to ensuring the quality and safety of agricultural products [16]. Strengthening the detection of the quality and bioactive substances of agricultural products helps provide consumers with safe, high-quality, and nutritionally rich products [17]. Exploring efficient post-harvest preservation technologies can maintain the quality of fruits and vegetables, extend the shelf life of agricultural products, and reduce post-harvest losses [18].

Agricultural nanotechnology has emerged in this context, covering several highly promising research fields [19]. Nanomaterials are widely used in nanobiology and gene delivery due to their small size, large surface area, biocompatibility, biodegradability, low toxicity, and low immunogenicity. Moreover, compared with traditional plant transformation methods, gene engineering methods based on nanoparticles have the advantages of low cytotoxicity, ease of operation (for example, no need for unnecessary cell wall removal), species independence, and the ability to deliver multiple biomolecules (such as nucleic acids, imaging agents, and regulatory active molecules), among others, opening up new avenues for the cultivation of excellent crop varieties and showing good application prospects in plant genetic engineering [20].

Developing nano-fertilizers and nano-pesticides, by optimizing their release mechanisms, can significantly improve the utilization efficiency of nutrients and pesticides and reduce the negative environmental impacts [21]. In addition, nanomaterials have shown great potential in adsorbing environmental pollutants, thus purifying soil and water bodies, which helps create a favorable ecological environment for agricultural production. Nanomaterials are one of the effective ways to degrade and remove environmental pollutants, and related research has made rapid progress in recent years. Carbon nanotubes, metal oxide nanoparticles, polymer nanoparticles, etc., have all been used to remove pollutants from water [22].

The unique physical and chemical properties of nanomaterials are utilized for the detection, control, and removal of pollutants in agricultural products, as well as for the analysis and detection of the quality and bioactive substances of products [17,23]. In the field of fruit and vegetable preservation, adding nanomaterials to the biopolymer substrate is considered the best choice. Technologies such as nanocomposite films can effectively inhibit the growth of microorganisms, delay the senescence of fruits and vegetables, and maintain their freshness and quality. The nanoscale endows materials with unique properties, including physical barrier properties, mechanical properties, and antibacterial properties, which can extend the shelf life of fruits and vegetables [24]. Some metal nanoparticles, such as silver nanoparticles (Ag NPs) [25], gold nanoparticles (Au NPs) [26], zinc nanoparticles (Zn NPs) [27], etc., are also used for fruit and vegetable preservation due to their own antibacterial properties and physical and chemical characteristics [28]. Agricultural nanotechnology is expected to provide innovative solutions to the global food security supply problem and promote the sustainable development of agriculture.

## 2. Preparation and Performance Regulation of Agricultural Nanomaterials

### 2.1. Classification and Preparation Methods of Nanomaterials

Currently, the nanomaterials in application mainly include organic nanomaterials such as cellulose nanoparticles, chitosan nanoparticles, and starch nanoparticles, as well as inorganic nanoparticles. Metals and metal oxide nanoparticles, for example, silver nanoparticles (Ag NPs), zinc oxide nanoparticles (ZnO NPs), titanium dioxide nanoparticles (TiO_2_ NPs), etc., are also among them [29].

#### 2.1.1. Inorganic Nanomaterials

Inorganic materials mainly include metals and metal oxides. Metal–organic frameworks (MOFs), a kind of porous material with a periodic network structure, are self-assembled from metal elements and organic ligands under strong coordination interactions [30]. Defect engineering refers to the use of technical methods, such as etching, to break the perfect periodic structure in the crystal and change the original properties of the material. A large number of research results show that introducing defects into MOFs’ structure is beneficial and can change their adsorption performance, catalytic activity, electrical conductivity, etc. [31].

The second is the liquid-phase exfoliation method. The liquid-phase exfoliation method can be used to exfoliate bulk materials into nanosheets in various ways, such as ultrasonic exfoliation and mechanical exfoliation, which is common in two-position stacked MOFs. Weak interactions, such as dielectric interactions, hydrogen bonds, and van der Waals forces, hold the layers of the multi-layer MOF together. If the in-plane bonding is stronger than these interactions, they can be exfoliated into two-dimensional (2D) nanosheet stacks by a top-down method [30].

**Figure 2:**
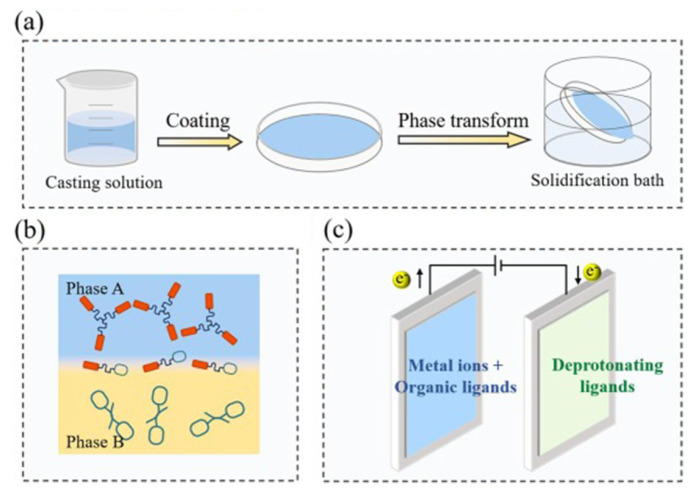
(**a**) Phase transformation. (**b**) Interfacial polymerization. (**c**) Electrochemical synthesis [32].

MOFs act as functional fillers in composite membranes and are usually mixed with cross-linking agents or selected high-strength base materials to form a film. According to the requirements of the selective separation membrane, it is usually necessary to design a membrane with high strength, multiple channels, small pore size, and rapid mass transfer. Currently, there are four methods commonly used for preparing MOF composite membranes: phase inversion method, interfacial polymerization method, solvothermal method, and electrochemical synthesis method (Figure 2) [32].

The aggregation and oxidation of Ag NPs affect their stability and antibacterial properties. Ag NPs can be mixed with other materials, including polyvinyl alcohol (PVA), chitosan (CS), and MgO, to produce Ag@composite materials with multiple functions, which can help solve these problems [33]. The one-pot chemical reduction synthesis method is adopted, in which silver nitrate and PANI are mixed in ethylene glycol at 80 °C for 30 min [34]. Sourav Kumar et al. used the sol–gel method to filter TiO_2_ powder and mixed it with silver nitrate for a whole day [35]. Zhao et al. synthesized Ag-MOF with a particle size of 2.0 nm using a mechanical method [36].

#### 2.1.2. Organic Nanomaterials

Electrospinning is a widely used technology for processing carbon nanotubes and their corresponding polymer nanocomposites. The main advantage of this method is that the fiber diameter and pore size can be controlled by modifying the setting parameters [37]. Chitosan is a polysaccharide that is often used as a biomaterial. Among these materials, chitosan and Ag NPs can be synthesized into an antibacterial and durable composite film through the method of physical stirring [38]. Additionally, multi-component nanocomposites based on chitosan, cellulose/chitosan nanofibers, and essential oils can be developed through the solution casting technique. Dissolving fertilizers in a chitosan nanomaterial solution and stirring them magnetically makes this process very simple [39].

### 2.2. Principles and Methods for Performance Enhancement

#### 2.2.1. Gene Delivery

In genetic engineering methods, engineered carbon nanomaterials have many excellent applications due to their outstanding mechanical, electrical, optical, and thermal properties. They mainly include carbon dots (CDs), graphene, graphene oxide, nanodiamonds, etc. The stable and fluorescent properties of CDs enhance their cellular uptake, transportation, and accumulation in plants. Using polyethyleneimine (PEI) as a carbon source, water-soluble CDs for DNA adsorption were successfully synthesized through a solvothermal reaction, and a low-pressure spray method was developed to silence the endogenous plant gene (magnesium chelatase) [40]. Through particle bombardment, nanodiamonds with high density and high hardness can be delivered into plant tissues. For every kind of nanoparticle, the most critical obstacle to penetrating plant cells is the cell wall. Single-walled carbon nanotubes can penetrate the cell wall, pass through the lipid bilayer surrounding the chloroplasts, and deliver nucleic acids into the chloroplasts. A mathematical Lipid Exchange Envelope Penetration (LEEP) model has been developed to explain the mechanism of subcellular uptake of nanoparticles in plants [41].

Magnetic nanoparticles (MNPs) are a very effective method for gene delivery. MNPs are usually iron oxides coated with biomolecules and are delivered to target cells by applying an external magnetic field [42]. Demirer et al. constructed an siRNA-SWNT delivery system using π–π stacking. The SWNT-based platform protects RNA from nuclease degradation and enables the effective delivery of RNA into intact plant cells, making it possible to silence the endogenous GFP gene in mutant plants [43].

#### 2.2.2. Crop Growth and Development

Nanomaterials regulate crop growth and help plants overcome and adapt to some adverse environments. One of the most important methods is the application of nano-fertilizers. One aspect in which nano-fertilizers are superior to traditional fertilizers is that nano-fertilizers can achieve controlled release of elements. In controlled-release nano-fertilizers, nutrients are encapsulated in carrier materials composed at the nanoscale, such as polymers, lipids, or inorganic compounds [44]. Environmental conditions such as temperature, pH value, and humidity, as well as stimulus-responsive mechanisms such as biodegradation or enzyme-mediated degradation, may change the release of nutrients from these carriers [45].

The formation of a complex between chitosan and fertilizer molecules leads to enhanced accessibility of the fertilizer to plants. Chitosan nanoparticles have the potential to serve as carriers for the regulated release of NPK fertilizers in the agricultural environment. Chitosan molecules are modified to release plant growth regulators, herbicides, insecticides, and fertilizers. Chitosan acts as a carrier and controlled-release matrix, protecting biomolecules from the effects of pH value, light, and harsh temperatures. In addition, it extends the duration of the release of active substances, thereby protecting plant cells from sudden release. It has been found that the use of chitosan-based nano-fertilizers can enhance the uptake of nutrients by plants [44].

#### 2.2.3. Detection and Removal of Pollutants

**Figure 3:**
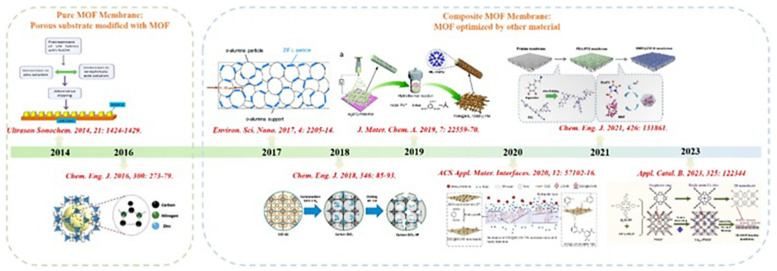
Development of MOF membranes for pollutant removal [32].

The strong metal-charged bonds between metal ions and negatively charged ligands in MOFs endow them with a regular structure and stable chemical bonds. Compared with previous amorphous porous organic materials, due to their highly developed pore structure and specific surface area, the properties of the pores can be controlled. By combining different types of polymers with oxide and carbon-based materials, new composite materials with stronger and more attractive electronic properties can also be fabricated [46]. In the field of pollutant removal, researchers have conducted extensive and meticulous explorations of both the preparation methodologies and modification technologies of MOF membranes (Figure 3).

The allotropes of carbon in carbon nanotubes (CNTs) are usually bonded together through strong van der Waals interactions. Therefore, they usually tend to aggregate, which reduces their surface area and leads to a larger mesoporous volume, which can be used as an adsorption site for accommodating organic pollutants(OPs).

#### 2.2.4. Detection of Quality and Bioactive Substances

The quality and safety of agricultural products are related to people’s life and health. In order to ensure the quality and safety of the supplied agricultural products, it is urgent to develop accurate detection methods. Currently, the detection methods for pesticide residues are mainly divided into two categories. One is the confirmatory detection technology, which mainly includes gas chromatography, liquid chromatography, gas chromatography–mass spectrometry, and liquid chromatography–mass spectrometry. The other is rapid detection technology, which is mainly based on the enzyme inhibition method, enzyme-linked immunosorbent assay (ELISA), and aptamer detection method [17].

Confirmatory detection technology has the advantages of high stability and good reproducibility, but it also has the disadvantages of high cost, long time consumption, and complex operation and cannot meet the needs of the quality and safety detection of fresh agricultural products in China. Therefore, rapid detection technologies such as the enzyme inhibition method and ELISA, which are easy to operate, low in cost, and short in time consumption, have attracted the attention of many researchers [17].

The most commonly used enzymes in the enzyme inhibition method and ELISA are natural biological enzymes. Natural biological enzymes are an important class of biological catalysts, and the vast majority of natural biological enzymes are composed of proteins. Because they are extremely easy to reduce and lose their catalytic activity in environments such as high temperature or too high or too low pH and are easily affected by the surrounding environment, the accuracy of the detection and analysis technologies using these biological enzymes is affected [47].

In order to make up for defects such as the poor tolerance of natural biological enzymes, researchers have developed nanozymes with the advantages of high environmental tolerance, wide applicability, simple preparation process, and low cost. In recent years, nanozymes have been increasingly widely used in fields such as the detection of agricultural pollutants (such as pesticides, heavy metals, and mycotoxins) [48], medical diagnosis [49], and antibacterial treatment [50].

The inherent physical and chemical properties of nanomaterials endow nanozymes with more possibilities. For example, by adjusting the morphology, structure, crystal plane, composition, etc., of nanoparticles, they can exhibit adjustable enzyme-like (oxidase and peroxidase) catalytic activities under different external environments, such as pH value, temperature, substrate type, and oxygen concentration. Moreover, the unique properties of nanomaterials, such as the magnetic effect and photothermal effect, endow them with a broader design space. The catalytic behavior of nanozymes can be affected by external factors such as light, heating, ultrasound, or a magnetic field.

Oxidase is one of the important classes of enzymes, and its reaction mechanism is to catalyze the oxidation of substrates in the presence of molecular oxygen (O_2_). Oxidases can be further classified according to the type of catalytic substrate, such as glucose oxidase, sulfite oxidase, etc. [51]. Peroxidases can decompose various peroxides (ROOH, H_2_O_2_) to oxidize substrates. Common peroxidases include horseradish peroxidase (HRP), cytochrome c peroxidase, etc. [52]. Peroxidase nanozymes are nanozymes that catalyze the oxidation of target substrates using H_2_O_2_ as an oxidant.

**Figure 4:**
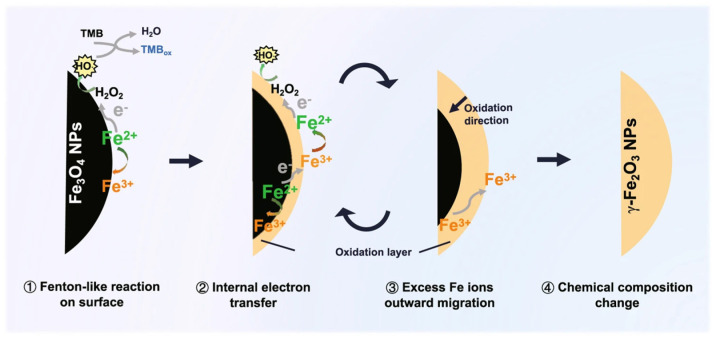
Schematic diagram of the catalytic mechanism of the peroxidase-like activity of Fe_3_O_4_ NPs [53].

Fe_3_O_4_ is one of the earliest discovered nanozymes. Many studies have proposed a surface Fe^2+^-induced Fenton reaction to explain their peroxidase activity. In 2022, it was pointed out that the Fe^2+^ inside Fe_3_O_4_ can transfer electrons to the surface through the Fe^2+^-O-Fe^3+^ chain, regenerate the surface Fe^2+^, and make the peroxidase catalytic reaction proceed continuously (Figure 4) [53].

Peroxidase nanozymes catalyze luminescent substrates such as 3,3′,5,5′-tetramethylbenzidine (TMB) and o-phenylenediamine to develop color, generating optical signals to achieve the detection of target substances, and have a wide range of applications in the detection of pesticides, mycotoxins, metal ions, etc. One researcher developed a turn-on-mode chemiluminescent detection method for organophosphorus. The principle is that ethion and ethanol compete for binding sites with Fe_3_O_4_ nanoparticles, inhibiting the activity of Fe_3_O_4_ nanozymes and reducing the chemiluminescence intensity to achieve the detection of ethion [54].

#### 2.2.5. Preservation of Fruits and Vegetables

The post-harvest preservation of fruits and vegetables is also an important part of ensuring product safety and quality. Since fruits and vegetables still have a certain cell respiration and metabolic function after harvest, they will consume nutrients such as sugars, leading to the release of endogenous ethylene, which accelerates the ripening and aging of fruits and vegetables and reduces their commercial quality. At the same time, the surface of fruits and vegetables is vulnerable to the invasion of harmful microorganisms, resulting in easy decay and deterioration during storage and transportation, causing quality deterioration [13].

Appropriate food packaging can protect fruits and vegetables from environmental and spoilage factors. Currently, commonly used preservation methods are divided into physical preservation methods and chemical preservation methods. However, due to the high energy consumption, high operation cost, strict equipment and technical requirements [55], and potential toxicity of chemical materials that may remain on the fruits [56], which potentially pose a risk to consumers’ health, the ability to use nanomaterials with improved properties to address these issues is a current research priority [57].

As an antibacterial agent, Ag NPs can fight against a variety of symbiotic and pathogenic strains such as fungi and viruses. They exert an antibacterial effect by binding to DNA, proteins, and enzymes and acting against metabolic activities [58]. Some people also combine organic and inorganic materials according to the complementarity of their physical and chemical properties to achieve a better preservation effect.

Sodium alginate/gelatin-based films have excellent film-forming properties and good compatibility but poor antibacterial performance. The performance of chitosan (CS) films will be significantly attenuated due to the interference of oxygen and water, and they have poor antioxidant capacity. Selenium nanoparticles (Se NPs) have good antibacterial properties, antioxidant activity, thermal stability, and low toxicity and can be used as an enhancer for nanocomposite films [59].

## 3. Application Progress

### 3.1. Gene Delivery

In recent years, there has been a keen interest in exploring the effects of CDs on plant growth, development, photosynthesis, and resistance to abiotic/biotic stresses [60]. Wang et al. successfully grafted PEI onto the surface of CDs, thereby endowing the CDs with a positive charge. This strategic surface modification of CDs facilitates their electrostatic interaction with plasmid DNA (pDNA), enabling highly efficient genetic transformation and subsequent protein expression in a diverse range of plant species, such as *Oryza sativa*, *Triticum aestivum*, and *Vigna radiata* [61].

**Figure 5:**
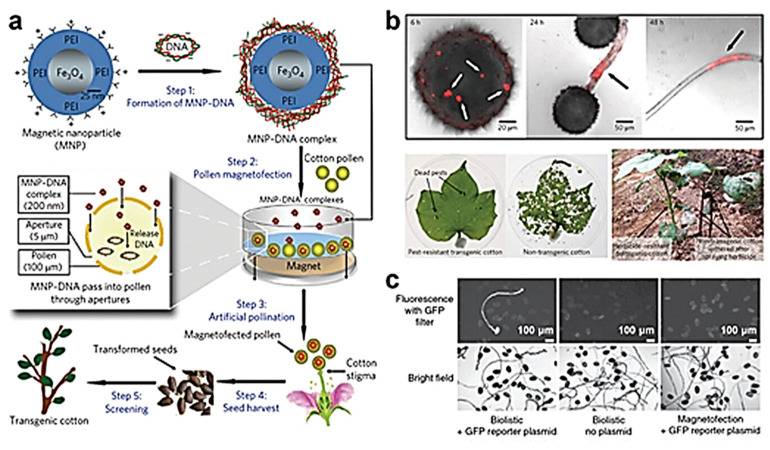
Pollen magnetotransfection using MNPs. (**a**) Pollen magnetotransfection consists of five steps: (1) formation of the MNP-DNA complex; (2) pollen magnetotransfection with cotton pollen; (3) magnetotransfected pollen grains are used for artificial pollination; (4) seed harvesting; (5) screening of transgenic plants. (**b**) Time-lapse tracking of fluorescent MNPs labeled with Lumogen F Red 305 in pollen grains and pollen tubes within 48 h. (**c**) There is no evidence of transient transformation by pollen magnetotransfection, while there is evidence of a combination of biolistic and GFP reporter plasmids [69].

Magnetic induction, defined as the delivery of nucleic acids guided by magnetic force, has been popularized in animal and medical systems [62], but its application in plants is rare. A research team has developed a simple and effective method to produce transgenic seeds using magnetic iron oxide without the need for tissue culture regeneration [63]. With the help of an external magnetic field, DNA-loaded MNPs are delivered into the pollen through the pores in the pollen wall. After pollination with magnetically infected pollen, transgenic plants were successfully obtained from the transformed seeds, in which the exogenous genes were stably integrated into the host genome (Figure 5a). Fluorescence microscopy observations revealed that MNPs were mainly distributed in pollen grains. They were transferred along the pollen tube within 48 h (Figure 5b).

Using this method, the authors developed four transgenic dicotyledonous plants, including cotton (*Gossypium hirsutum* L.), pumpkin (*Cucurbita moschata*), pepper (*Capsicum annuum* L.), and zucchini (*Cucurbita pepo* L.), and a monocotyledonous plant, lily. This work combines magnetofection technology with pollen-mediated genetic transformation technology to establish a pollen magnetofection technology system. This system does not require a complex regeneration process and is independent of the host. In addition, pollen magnetofection technology can improve the genetic transformation efficiency, shorten the breeding time, and achieve high-throughput screening and co-transformation of multiple genes and is of great significance for accelerating the breeding of new transgenic plant varieties.

However, the magnetofection of nucleic acids into pollen through the pores in the pollen wall is still controversial. Some studies have found that in two monocotyledonous plants, maize and sorghum, transient transformation through pollen magnetofection has not been achieved (Figure 5c) [64]. For example, it has been reported that exogenous genes loaded on starch particles (50–100 nm) were successfully delivered into suspension cells with the assistance of ultrasonic vibration. Starch nanoparticles loaded with DNA are very stable against nucleases, but ultrasonic waves can damage plant cells, and the genetic transformation efficiency of starch nanoparticles is relatively low [65,66].

Calcium phosphate (CaP) is an ideal carrier for exogenous genes in animal cells. Its transfection efficiency is even higher than that of commercial transfection reagents [67]. Calcium is an essential macronutrient for plants, which enters plant cells through Ca^2+^-permeable ion channels on the plasma membrane and acts as a second messenger in many developmental and physiological processes. Some researchers have studied the tissues and cells of mustard (*Brassica juncea* L.). They found that the transformation efficiency of DNA/CaP was as high as 80.7%, which was significantly higher than that of traditional Agrobacterium and naked DNA. However, the genetic transformation efficiency of CaP for chicory was only 10%. It is speculated that the difference in genetic transformation efficiency among different plants may be due to the different pore sizes of the cell walls [68].

### 3.2. Nano-Fertilizers and Chemical Fertilizers

While traditional chemical fertilizers enhance agricultural productivity, their excessive application has a negative impact on soil health, resulting in low nutrient utilization efficiency and environmental pollution [70]. Therefore, it is extremely urgent for modern agriculture to search for new materials and technologies [45]. In recent years, nanotechnology has developed rapidly, providing eco-friendly approaches in aspects such as nano-fertilizers, nano-insecticides, nano-seed priming, and stress tolerance.

According to different nutrient elements, nano-fertilizers are generally classified into macronutrient nano-fertilizers and micronutrient nano-fertilizers. Macronutrient nano-fertilizers are combinations of nanomaterials with macronutrients such as nitrogen (N), phosphorus (P), and potassium (K) to achieve controlled and slow release of nutrients [3]. Currently, multiple nutrient elements are encapsulated in specific nanomaterials to provide a series of nutrients for crops. For example, the nano-fertilizer composed of urea coated with hydroxyapatite nanomaterials replenishes nitrogen and phosphorus for rice with a lower application rate. Using half of the recommended dose of this nano-fertilizer can achieve the same crop yield as the recommended dose of commercial urea [71]. Some studies have found that foliar application of 50% NPK nano-fertilizer can improve the yield and quality of potatoes [72]. Research has shown that the application of both conventional fertilizers and nano-fertilizers can enhance the quality and yield of rice. The application of nano-nitrogen, phosphorus, and potassium (nNPK) and a combination of conventional nitrogen, phosphorus, and potassium (NPK) + nNPK significantly improves the milling quality, elongation rate, and gelatinization temperature of rice, while reducing the gel consistency, amylose content, and milled rice yield [73]. It has been found in some studies that zinc ZnO NPs can improve the germination of peanut seeds [74]. Ag NPs enhance the germination potential of rice seeds by increasing the activity of α-amylase [75]. After the application of copper nanoparticles (Cu NPs), the seed germination in tomatoes and lettuce has been improved [75]. Under abiotic stress, nano-fertilizers of micronutrient elements can promote the growth of crops. However, due to differences in concentration and type, their impacts on plants also vary (Table 1).

In addition to promoting crop growth, the application of nanoparticles can also enhance the stress tolerance of plants (Table 2). CeO_2_ NPs reduce membrane lipid peroxidation and oxidative damage by regulating the antioxidant enzyme system as well as the levels of proline and phytohormones [7]. Some studies have shown that nanoparticles (such as SiO_2_, ZnO, Se, and graphene) can mitigate the adverse effects of cold damage in sugarcane by maintaining the photochemical efficiency of photosystem II (PSII), photooxidizable photosystem I (PSI), and photosynthetic gas exchange [76]. Some researchers have found that titanium dioxide nanoparticles (TiO_2_ NPs) can reduce the incidence of spot disease in wheat caused by Bipolaris sorokiniana [4]. Similarly, the application of Ag NPs (5 μg/mL) leads to a 70–80% reduction in black spot disease in Arabidopsis thaliana caused by Alternaria brassicicicola [5]. Ag NPs can also increase the aboveground mass of sunflowers, reduce the levels of H_2_O_2_ and malondialdehyde (MDA), alleviate the oxidative damage caused by salt stress, and enhance the stress resistance of sunflowers [6].

Fungicides encapsulated in small-dose lignin nanoparticles can effectively control trunk diseases of grapevines [77]. Additionally, some nano-fertilizers can alter the activity of traditional chemical pesticides, enhancing the effectiveness of plant pest control. A study has found that when cationic nano-chitin whiskers (NC) are applied in combination with chemical pesticides, the corrected mortality rate of wheat aphids can be maintained above 95%, and the usage amount of chemical pesticides can be reduced [78].

Controlled release of metal nanoparticles is crucial for prolonging their antibacterial activity. Therefore, mixtures of biopolymers and nanoparticles are used as antimicrobial agents and nano-insecticides to meet agricultural demands. However, some scholars express concerns about the safety of nanomaterials. They believe that a large number of nanoparticles in agriculture may disrupt the nitrogen cycle. Research has shown that, based on the ^15^N atom excess rate, different concentrations of Ag NPs significantly inhibit the conversion of ^15^N-labeled NH_4_^+^ to NO_3_^−^. Ag NPs suppress the activity of nitrogen cycle enzymes, the number of nitrifying bacteria, and the total nitrification rate [79]. Thus, the successful application of nanotechnology in sustainable agriculture lies in dispelling concerns and gaining wide public acceptance.

### 3.3. Nano-Sensors

Recently, electrochemical nano-sensors utilizing carbon nanotubes and hematite nanostructures have been developed, which can quickly and accurately measure the nitrate content in field soil [80]. Research has proven that machine learning technology can be used in combination with portable nano-sensors made of screen-printed electrodes (SPEs) modified by composite nanomaterials to predict the carbendazim (CBZ) residues in rice and tea with high accuracy and precision [81]. In addition, gas nano-sensors such as polyaniline and Ag cantilever nano-sensors can quickly identify the presence of insects in farmland by reducing the resonance frequency in the presence of male volatiles (pheromones), providing an effective means for precise pest management [82]. Compared with traditional sensors, these nano-sensors have higher sensitivity and stability, portability, and a longer service life, providing the necessary technical support for the future agricultural revolution. Nanotechnology improves the efficiency of agricultural output by reducing the excessive use of chemical fertilizers and pesticides. The controlled release of metal nanoparticles is the key to prolonging their antibacterial activity. Therefore, a mixture of biopolymers and nanoparticles is used as an antimicrobial agent and nano-insecticide to meet agricultural needs. However, some scholars are concerned about the safety of nanomaterials, believing that a large number of nanoparticles in agriculture may disrupt the nitrogen cycle. Some research has found that according to the excess rate of 15N atoms, different concentrations of Ag NPs have an obvious inhibitory effect on the conversion of 15N-labeled NH_4_^+^ to NO_3_^−^. Ag NPs inhibit the activity of nitrogen cycle enzymes, the number of nitrifying bacteria, and the total nitrification rate [79]. Therefore, the successful application of nanotechnology in sustainable agriculture lies in dispelling concerns and gaining widespread public recognition.

### 3.4. Removal of Pollutants

With the emergence of nanomaterials, the application of nano-adsorbents in wastewater has been widely explored. Nanotechnology, as an emerging technology dedicated to targeting specific activities, has been widely applied in the field of environmental analysis, including environmental remediation, pollutant detection, desalination of seawater, and other aspects [16]. Nanomaterials with high surface area and nano-sized pores play an important role in the development of new adsorbents for wastewater treatment, mainly including MOFs and carbon-based nanomaterials.

Some researchers have synthesized superparamagnetic Fe_3_O_4_@Al-based metal–organic framework (Fe_3_O_4_@Al MOF) nanocomposites and found through research that Fe_3_O_4_@Al MOF has a very good adsorption effect on Congo red and is a promising material for its removal [83]. Graphene (GS) is defined as a single layer of sp^2^ allotropic carbon atoms arranged in a two-dimensional hexagonal honeycomb lattice structure. GS has been widely used to adsorb various organic pollutants (OPs), such as dyes, antibiotics, oils, and pesticides [84]. Some studies have found through theoretical calculations that the interaction between the surface of GS and chlorinated pesticides is very strong, and GS can adsorb chlorinated pesticides in water [85]. Nanoparticles have been subjected to functionalization treatments. For example, their electron density and specific surface area have been increased. This can enhance the nanoparticles’ adsorption capacity for pollutants and contribute to the removal of harmful substances such as dyes, radionuclides, and heavy metals (Table 3) [86].

### 3.5. Detection of Quality and Safety

The production and use of chemicals have led to an increase in chemical pollutants in the environment, such as pesticides and heavy metal ions. The enrichment of pollutants in agricultural products may ultimately pose a serious threat to human health. Therefore, pesticide residues can be detected in various consumer products, including dairy products, fruits, and vegetables [11]. Researchers have found that nanozymes based on molybdenum, gold, cerium, etc., have oxidase activity, and molybdenum-based, gold-based, and cerium-based nanomaterials all play an important role in the rapid detection of mycotoxin residues in agricultural products [52].

Similarly, many nanomaterials such as germanium dioxide (GeO_2_), nickel oxide (NiO), gold (Au), and platinum (Pt) also have peroxidase activity and are widely used in the detection field. By using an alkaline phosphatase–manganese dioxide (MnO_2_) oxidase-like tandem catalytic amplification system to detect ochratoxin A (OTA) in grape juice at trace levels, it was found that the catalytic activity of MnO_2_ can last for 60 days, demonstrating that MnO_2_ nanozyme has stronger environmental tolerance [88]. Chalcopyrite (CP) is a widely distributed copper–iron sulfide mineral, and CP nanoparticles (CP NPs) exhibit strong peroxidase-like activity. Due to hydrogen atom transfer and single-electron transfer of ascorbic acid, glutathione, and cysteine, the activity of CP nanozyme is inhibited to generate free radicals. The total antioxidant capacity probe based on the CP NP enzyme has been successfully used to detect the total antioxidant capacity in citrus fruits [89].

It has been found in research that Au NPs can detect mycotoxins in corn, and metal ions with peroxidase-like properties have played a huge role in rapid detection [90]. After modifying the surface of Au NPs with a K^+^ aptamer that reduces catalytic activity, the color development of 3,3′,5,5′-tetramethylbenzidine (TMB) can be enhanced. This can achieve the rapid detection of K^+^ [91].

### 3.6. Preservation of Fruits and Vegetables

Vegetables and fruits are prone to deterioration and decay during the processes of harvesting, storage, and transportation. Therefore, the development of environmentally friendly and multifunctional fruit and vegetable preservation materials has become a major focus in the fields of food science and production [18]. Currently, the nanomaterials applied to the preservation of fruits and vegetables mainly include organic nanomaterials such as cellulose nanoparticles, chitosan nanoparticles, and starch nanoparticles, as well as inorganic nanoparticles, for example, silver nanoparticles (Ag NPs), ZnO NPs, titanium dioxide nanoparticles (TiO_2_ NPs), etc. [29].

**Figure 6:**
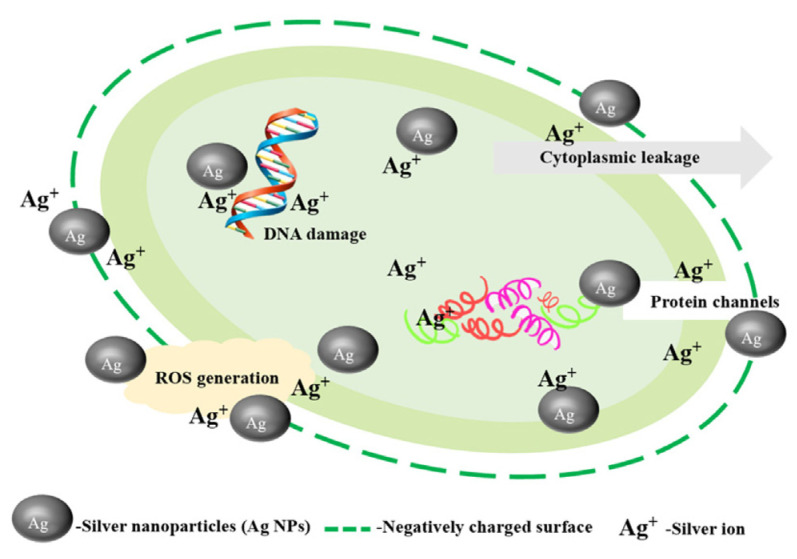
The nanoscale and ionic properties of silver nanoparticles (Ag NPs) determine the mechanism of their bactericidal activity [97].

Strawberries have a high softening rate and are vulnerable to fungal infection, leading to spoilage. To extend the shelf life of strawberries and reduce post-harvest losses, a composite film made of Ag NPs, chitosan, and polyvinyl alcohol has been used for strawberry preservation. Research shows that this composite film has excellent mechanical properties and antibacterial activity. The antibacterial properties of Ag NPs have played a crucial role (Figure 6). Strawberries covered with this film show no signs of decay after being stored at 25 °C and 50% relative humidity for 9 days, which greatly extends their shelf life [37].

Sodium alginate/gelatin-based films have excellent film-forming properties and good compatibility, but their antibacterial performance is poor. The performance of chitosan (CS) films will be significantly attenuated due to the interference of oxygen and water, and they have poor antioxidant capacity. Selenium nanoparticles (Se NPs), with good antibacterial properties, antioxidant activity, thermal stability, and low toxicity, can serve as an enhancer for nanocomposite films. Some research has prepared a chitosan–andrographis paniculata extract–selenium nanoparticle (CS-APE-Se) composite film by combining Se NPs, chitosan, and andrographis paniculata extract and used it for strawberry preservation. The study found that covering strawberries with the composite film containing Se NPs effectively prevents physical damage to strawberries and extends the preservation time of strawberries by 10 days [59].

Polyvinyl alcohol (PVA), due to its hydrophilicity and excellent film-forming ability, can be applied to the preservation of fruits and vegetables. However, it has poor mechanical strength during the swelling process, which limits its application. Au NPs, with good biocompatibility and antibacterial properties, are combined with PVA to prepare a film to overcome these limitations. The results show that the addition of Au NPs improves the mechanical properties and antibacterial performance of the film and inhibits the growth of microorganisms on the surface of bananas, and the preservation effect is better than that of the PVA film [92].

TiO_2_ NPs are widely used in the preservation of fruits and vegetables due to their excellent biocompatibility and photocatalytic and antibacterial properties [93]. A biodegradable film based on chitosan–alginate–TiO_2_ was prepared by adding different concentrations of TiO_2_ NPs, and its effect on the storage quality of tomatoes was studied. The study shows that the addition of TiO_2_ NPs increases the ultraviolet barrier property of the film by 88.6%. Using this film to preserve tomatoes extends the shelf life to 8 days, and the pH value, soluble solids content, and mass of tomatoes are also stable, with no bacterial growth [94].

Some researchers have prepared a new type of nanocomposite film based on gum arabic (GA) and cellulose nanocrystals (CNC) and studied its preservation effect on strawberries. The results show that after storing at 50% relative humidity and 4 °C for 9 days, the weight loss rate of strawberries is 23.80% lower than that of the control group [95]. Bacterial nanocellulose (BNC), when applied as a biological material in preservation films, can improve their physical and mechanical properties, thus extending the shelf life of fruits and vegetables. A bacterial nanocellulose structure (SFMPI-BNC) film was prepared using the matrix of protein isolate extracted solely from sunflower meal (SFMPI) for strawberry preservation. The study shows that compared with the SFMPI film, using the SFMPI-BNC film to package strawberries results in a slower change in the physical and chemical characteristics of strawberries [96].

## 4. Conclusions and Prospects

In recent years, nanotechnology has witnessed rapid development, with significant progress also achieved in its agricultural applications. For instance, in the field of genetic engineering, magnetofection technology has been developed, utilizing nanomaterials as gene delivery vectors. Research and applications of nano-fertilizers and nano-pesticides, as well as the use of nano-sensors for precise field management, have emerged. Nanosomaterials can adsorb organic pollutants in water. Nanozymes have increasingly broad applications in detecting the quality and contaminants of agricultural products, addressing the drawback of poor tolerance of natural enzymes. In terms of fruit and vegetable preservation, nanocomposite fresh-keeping films have been developed, circumventing the high-energy consumption of traditional methods.

Despite these advancements, large-scale application of agricultural nanotechnology still faces numerous challenges to overcome, primarily in technical, economic, and safety aspects. In genetic engineering, nanoparticle-based delivery systems exhibit significant differences in transformation efficiency across different plant species, and the delivery efficacy of some materials is limited by their inherent properties, requiring overall improvements in technical maturity. Large-scale application of nano-fertilizers is constrained by complex production processes and difficulties in transportation and storage. In pollutant treatment, practical application of nanomaterials still needs to address issues such as high operational costs and imperfect environmental compatibility assessment systems. When using nanozymes for rapid detection, enhancing detection stability and reducing costs are essential to promote their widespread application in real-world scenarios. The application of nanocomposite fresh-keeping films is hindered by complex material regulation, incomplete safety evaluation systems, and limited application scenarios.

Future research should focus on breakthroughs in the following directions: developing technologies with higher efficiency and lower costs to facilitate large-scale application; designing biodegradable and environmentally friendly novel nanomaterials to reduce ecological risks; conducting interdisciplinary analyses of the impacts of nano-agrochemicals on food chains and ecosystems to determine their environmental effects; and improving safety evaluation systems and industry standards for nanomaterials to promote technological industrialization. This article elaborates on the progress of the application of nanotechnology in the agricultural field and horticultural crops from several aspects, including gene delivery, regulation of crop growth, adsorption of pollutants, quality and safety detection of agricultural products, and preservation of fruits and vegetables.

## Figures and Tables

**Table 1:** Effects of different micronutrient nano-fertilizers on various crops under abiotic stress. Abbreviation: ROS, reactive oxygen species.

Type	Conc.	Crop	Effect
Fe	50, 500, 2000 mg/L	Cucumber	Dose-dependent effects on biomass and antioxidant enzymes
Fe	10, 20 mg/L	Lettuce	Reduced growth and chlorophyll contents, and increased antioxidant enzyme activities
Fe	30–60 ppm	Garden pea	Improved seed mass and chlorophyll content
Cu	0, 100, 500 mg/L	Squash	Higher ionic Cu found in media amended with bulk Cu than with nCu
Cu	130, 660 mg/Kg	Lettuce	Increased shoot/root length ratio
Cu	0, 10, 20 mg/L	Lettuce	Negative effects on nutrient content, dry biomass, water content and seedling growth
Cu	0–1000 mg/L	Cucumber	Reduced growth and increased antioxidant enzymes
Cu	10–1000 mg/L	Radish, grasses	DNA damage, growth inhibition
Cu	50–500 mg/L	Tomato	Improved fruit firmness and antioxidant content
Cu	0, 20, 80 mg/Kg	Cilantro	Reduced germination and shoot elongation
Cu	100, 250, 500 ppm	Bean	Growth inhibition and nutrition imbalance
Cu	100–500 mg/L	Garden pea	Reduced plant growth and enhanced ROS production and lipid peroxidation
Zn	1000 mg/Kg	Cucumber	Root tip deformation and growth inhibition
Zn	500 mg/Kg	Garden pea	Decreased chlorophyll and H_2_O_2_ contents
Zn	1000 mg/L	Spinach	Growth reduction
Zn	1 mg/mL	Tomato, eggplant	Reduced fungal disease
Zn	100, 200, 500 ppm	Chili pepper	Improved germination
Zn	0–400 mg/Kg	Coriander	Improved pigment contents and defense responses
Zn	5, 10, 20 mg/L	Onion	Inhibition of root growth

**Table 2:** Effects of different nanomaterials on various crops under stress conditions. Abbreviations: MDA, malondialdehyde; MWCNT, multi-walled carbon nanotube; SWCNT, single-walled carbon nanotube; NP, nanoparticle; ROS, reactive oxygen species; SOD, superoxide dismutase.

Application Method	Type	Concentration	Plant Species	Type of Stress	Effects
Pre-sowing	MWCNT	10, 20, 40, 60 mg/L	Cabbage	Salinity	Increased growth, water uptake and net assimilation of CO_2_. Induced alterations in lipid rigidity, composition and permeability in the root plasma membranes
Pre-sowing	SiO_2_	0, 1.5, 3.0, 4.5, 6.0, 7.5 g/L	Pumpkin	Salinity	Enhanced seed germination, growth, photosynthetic parameters and antioxidant enzyme activity. Reduced MDA, H_2_O_2_, chlorophyll degradation and oxidative damage
Pre-sowing	Nano-silicon	10 mg/L	Hollyhock	Salinity	Increased germination, plant height, relative water content, fresh and dry weights, relative growth rate, total soluble sugars and membrane stability
Pre-sowing	Ag	0, 40, 80, 120 mg/L	Saffron	Flooding	Increased root growth, dry leaf weight and root length
Pre-sowing	Nano-selenium	1, 4, 8, 12 μM	Ajwain	High and low temperature	Improved plant growth, chlorophyll and leaf relative water content
Post-sowing	TiO_2_	0.05, 0.1, 0.2 g/L	Tomato	Heat stress	Enhanced photosynthesis by regulating energy dissipation and caused cooling of leaves through inducing stomatal opening
Pre-sowing	CuO, Al_2_O_3_, TiO_2_	0, 20, 200, 2000 μg/mL	Onion	Oxidative stress	Induced chromosomal aberration effect SOD and POD activities as well
Pre-sowing	SiO_2_	0.05, 0.5, 1.5, 2, 2.5 mg/L	Tomato	Salinity	Up-regulated the expression profile of salt stress genes (AREB, TAS14, NCED3 and CRK1).
Pre-transplanting	Ag	10, 20, 40 mg/L	Tomato	Salinity	Negatively affected the plant height, number of branches and fruit traits (diameter, weight and number of fruits).
Post-transplanting	Si	1, 2, 4, 5 cm^3^/L	Chili pepper	Salinity	Significantly regulated the plant to endure salt stress.
Post-transplanting	Nano-Ca	0.5, 1, 2, 3 g/L	Tomato	Salinity	Lower level significantly reduced the negative effects of salinity
Post-transplanting	Monopotassium phosphate, nano-calcium	0.5, 0.75, 1 g/L	Tomato	Salinity	Medium concentration of NMs improved stem diameter and number of flowers.
Post-transplanting	Nano-silicon	0, 1, 2 mM	Tomato	Salinity	Improve fresh weight, chlorophyll concentration, photosynthetic rate and leaf water content
Foliar application	Nano-silicon	1, 2 mM	Peregrina	Salinity	Enhanced vegetative parameters and chemical composition, decreased accumulation of Na, Cl and total phenolics and flavonoids in leaves
Foliar application	Ag	0.4, 40 mg/plant	Cucumber	Oxidative stress (nanotoxicity)	Enhanced respiration, inhibited photorespiration and reduced inorganic nitrogen fixation.

**Table 3:** Various nanoadsorbents used for remediation process [87].

Nanoadsorbent	Target Heavy Metal	pH	Temp °C	Time min	Dose	Adsorption Capacity mg/g
DMSA@Fe_3_O_4_ MNRs	Pb^2+^	5	28	60	0.1 g/L	46.18
Activated carbon prepared from apple peels (ACAP)	Cr (VI)	2	28	4 h	0.05 g/50 mL	36.01
Titanium dioxide nanoparticles with nano-zero-valent iron (nZVI)	Nitrate	4.185	–	150.09	10	0.982 g/L
Magnetic activated carbon (MAC)	Pb^2+^ and Cd^2+^	6 and 5	–	30	0.2 g/L	49.8, 86.2
Polypyrrole–iron oxide–seaweed nanocomposite	Pb^2+^	5	40	20	0.5 mg/g	97.25
Magnetic-activated carbon nanocomposite (mFe_3_O_4_@ACCs)	Pb (II)	11	–	15	40 mg	239
Activated carbon/nanoclay/thiolated graphene oxide nanocomposite (AC/NC/TGO)	Pb (II)	5	–	40	0.5 g/L	208
Magnetite-polyrhodanine core–shell nanoparticles	Hg^2+^	6.5	25	5 h	10 g/L	29.14
Chitosan–iron oxide (CS–Fe_2_O_3_) nanocomposite	Pb^2+^, Cd^2+^	6	50	180	0.01 g	214.923, 204.318
Nickel ferrite/titanium oxide magnetic nanocomposite(FeNi_3_/TiO_2_ nanocomposite)	Cr^6+^	3	–	15	500 mg/L	0.998
Thiol-lignocellulose sodium bentonite (TLSB) nanocomposites	Zn^2+^, Cd^2+^, Hg^2+^	4.84	40	40	0.05	357.29, 458.32, 208.12
Chitosan/multiwalled carbon nanotube (MWCNT)/PVA membrane composites	Pb^2+^	7	RT	300	0.5	35.148
Polyacrylamide/Sodium Montmorillonite (PAM/Na-MMT) Nanocomposites	Co^2+^ and Ni^2+^	6	–	–	0.1	98.67, 99.30
Magnetic Mesoporous Calcium Carbonate-Based Nanocomposite	Pb^2+^, Cd^2+^	5.5	–	12 h	0.2 g/L	1179.821
